# Salicin-7-sulfate: A new salicinoid from willow and implications for herbal medicine

**DOI:** 10.1016/j.fitote.2018.02.009

**Published:** 2018-06

**Authors:** Clarice Noleto-Dias, Jane L. Ward, Alice Bellisai, Charlotte Lomax, Michael H. Beale

**Affiliations:** Department of Computational and Analytical Sciences, Rothamsted Research, West Common, Harpenden, Hertfordshire AL5 2JQ, UK

**Keywords:** Salicin, Salicin-7-sulfate, Salix, Willow

## Abstract

Willow (*Salix* sp.) is a historically well-known herbal medicine that provided the lead compound (salicin) for the discovery of aspirin, one of the most successful plant derived drugs in human medicine. During a metabolomics screen of 86 *Salix* species contained in the UK National Willow Collection, we have discovered, isolated and fully characterised a new natural salicinoid – salicin-7-sulfate. This molecule may have important human pharmacological actions that need to be considered in determining the efficacy and safety of willow herbal medicines.

## Graphical abstract

**Figure:**
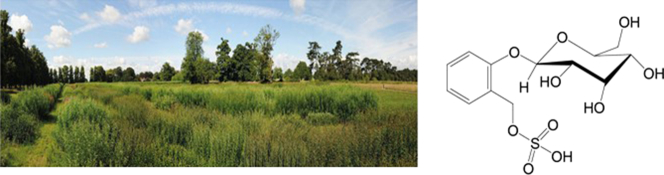

## 1 Introduction

Comminuted or powdered barks from *Salix* (willow) species, especially *S. alba, S. nigra, S. purpurea, S. daphnoides* and *S. fragilis* are well-known phyto-medicines with a history of ethno-medical use that stretches back to ancient Greek, Assyrian and Egyptian civilisations. The story of the identification of salicin **1** as an active analgesic from willow, and the introduction of the synthetic analogue aspirin (acetyl salicylate) **2**, which was to become a huge pharmaceutical success, has been well documented [1,2]. Both salicin and aspirin act as pro-drugs, being metabolised in humans to salicylate - the active pharmacophore that competitively inhibits cyclooxygenase [3,4], whilst aspirin itself also has a more direct action on cyclooxygenase, via irreversible acetylation of the active site [3].

The broad Salicaceae woody plant family contains several hundred species of *Salix* as well as the smaller *Populus* genus (e.g. poplar, aspen, cottonwoods). The family is characterised by the presence of phenolic glycosides, including, in many cases, the salicinoid sub-group of which salicin **1** represents the basic structure in a modular array of more complex analogues [5]. Possibly because of the success of aspirin, the potential for the discovery of new pharmacologically active compounds in the Salicaceae has been largely unexplored, although it has been suggested recently that the bioactivity of herbal extracts of willow cannot be accounted for by the levels of salicin alone [6]. In addition to pain relief, the use of aspirin in mitigation of thrombo-embolism is also well established and, more recently, both salicin and aspirin have been investigated for the prevention of cancer [[7], [8], [9], [10]].

**Fig. 1:**
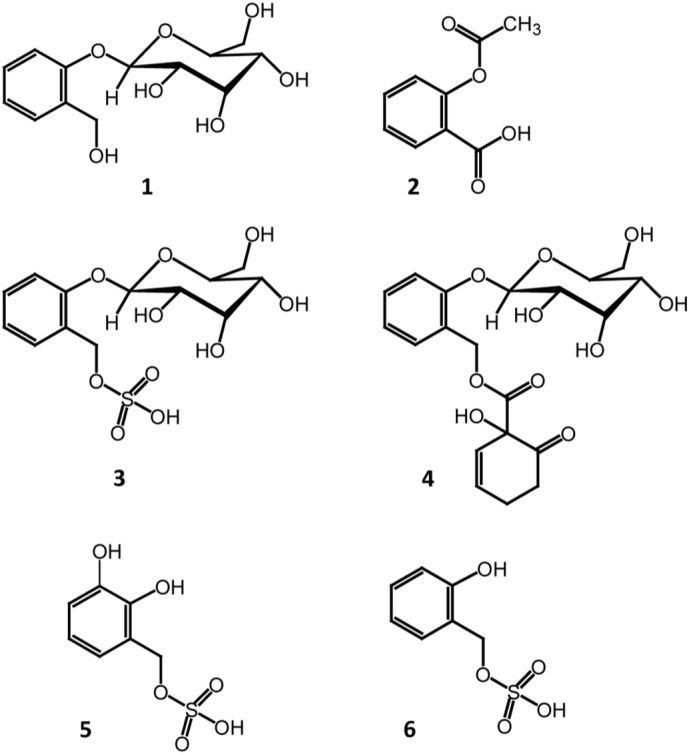
Chemical structures described in this paper.

As part of a programme dedicated to high value products from plants we have focussed on novel phytochemistry in the Salicaceae, in particular, those species contained in the 1500+ National Willow Collection (NWC), maintained as a short-rotation coppice plantation at Rothamsted Research. Taking a metabolomics approach [11] to polar extracts using NMR and high mass accuracy LC-MS-MS we have constructed a large annotated data-resource that spans the NWC and the full breadth of phenolic glycoside diversity. In this paper, we report on the discovery and structure determination of salicin-7-sulfate **3** (Fig. 1), a close analogue of salicin **1**, that potentially has a different metabolic fate in humans and thus requires further investigation in the context of efficacy and safety of the herbal materials.

## 2 Materials and methods

### 2.1 General experimental procedures

^1^H-1D and ^1^H-^1^H & ^1^H-^13^C 2D-NMR spectra were acquired on a Bruker Avance 600 MHz NMR spectrometer (Bruker Biospin, Germany), operating at 600.05 MHz for ^1^H and 150.9 MHz for ^13^C NMR spectra, using a 5 mm selective inverse probe. 1D ^1^H spectra were collected using 128 scans and by using the zgpr pulse sequence with a 90° angle. The residual HOD signal was suppressed by pre-saturation during a 5 s delay. Spectra consisted of 64,000 data points with a spectral width of 12 ppm. FIDs were automatically Fourier transformed using an exponential window function with a line broadening of 0.5 Hz. Phasing and baseline correction were carried out within the instrument software. 2D COSY, HSQC and HMBC spectra were collected using standard Bruker parameter sets and acquisition details are given in Supporting Information. All spectra were collected at 300 °K in D_2_O:CD_3_OD (8:2) and chemical shifts are given in δ, relative to d_4_-TSP [(trimethylsilyl) propionic acid, 0.01 % w/v] added as a chemical shift reference standard. NMR data was processed using TOPSPIN v. 2.1 (Bruker Biospin, Germany), MestReNova v. 6.0.2 (Mestrelab Research SL, Spain) and ACD NMR Processor (ACD Labs, Toronto, Canada)

UHPLC–MS were recorded with a Dionex UltiMate 3000 RS UHPLC system, equipped with a DAD-3000 photodiode array detector, coupled to an LTQ-Orbitrap Elite mass spectrometer (Thermo Fisher Scientific, Germany). UHPLC separation was carried out using a reversed-phase Hypersil GOLD™ column (1.9 μm, 30 × 2.1 mm i.d. Thermo Fisher Scientific, Germany) which was maintained at 35 °C. The solvent system consisted of water/0.1% formic acid (A) and acetonitrile/0.1% formic acid (B), both Optima™ grade (Thermo Fisher Scientific, Germany). Separation was carried out for 40 min under the following conditions: 0–5 min, 0% B; 5–27 min, 31.6% B; 27–34 min, 45% B; 34–37.5 min, 75% B. The flow rate was 0.3 mL/min, and the injection volume was 10 μL. Mass spectra were collected using an LTQ-Orbitrap Elite with a heated ESI source (Thermo Fisher Scientific, Germany). Mass spectra were acquired in negative mode with a resolution of 120,000 over *m/z* 50–1500. The source voltage, sheath gas, auxiliary gas, sweep gas and capillary temperature were set to 2.5 kV, 35 (arbitrary units), 10 (arbitrary units), 0.0 (arbitrary units) and 350 °C, respectively. Default values were used for other acquisition parameters. Automatic MS–MS was performed on the four most abundant ions and an isolation width of *m/z* 2 was used. Ions were fragmented using high-energy C-trap dissociation with a normalised collision energy of 65 and an activation time of 0.1 ms. Data was collected and inspected using Xcalibur v. 2.2 (Thermo Fisher Scientific, Germany). Data were analysed with the SIEVE™ 2.0 software (Thermo Fisher Scientific) using the Chromatographic Alignment and Framing algorithm. Frames were calculated from 0 to 40 minutes, between m/z 50 and 1500. Framing parameters were set at frame width of 2.5 minutes and *m/z* of 100 ppm, and peak intensity threshold of 2682520.

Compound isolation was carried out using an HPLC system (Dionex UltiMate 3000, Thermo Fisher Scientific) equipped with an Ascentis C-18 column (5 μm, 5 × 250 mm i.d., Supelco, UK) maintained at 25 °C. The chromatographic separation was performed by using a constant flow rate of 1 ml/min of the mobile phases water (A) and acetonitrile (B), both containing 0.1% formic acid. The binary gradient was: 10 min, isocratic of 2% B; 10 to 30 min, linear from 2 to 5% B, followed by 15 min of 5% B. Peaks were detected using wavelengths of 210 to 310 nm and the peak corresponding to salicin-7-sulfate was collected, in automation, by time (37.5–42 min) into glass tubes. Eighteen injections (100 μL each) were performed and fractions from repeated runs were combined and the solvent evaporated using a Speedvac concentrator (Genevac, Suffolk, UK).

Optical rotation was measured in water on an Anton Paar MCP-100 polarimeter using a 100 mm sample cell.

### 2.2 Plant material

Multiple dormant stems were harvested in February 2015 from the National Willow Collection (NWC) maintained at Rothamsted Research, Harpenden, UK (RRes), UK. Plants had been previously coppiced in February 2014. Each plot of the collection contains 10 plants that were generated from separate stem cuttings. Stem tissue portions (10–15 cm) were harvested from the top of each plant in a plot and combined to give a single sample. Tissue was kept at −80 °C prior to freeze-drying to remove residual water. After lyophilisation plant material was milled to a fine power (Ultra Centrifugal Mill ZM200, Retsch, UK). Milled tissue was maintained at −80 °C until analysis. Voucher specimens of lyophilised material have been retained and are available on request.

### 2.3 Metabolite extraction and isolation

For initial metabolite profiling by NMR and UHPLC-MS triplicate aliquots of milled freeze-dried willow stem powder (30 mg) were extracted as previously described [11]. Separate extractions were made for each analytical method. For compound isolation freeze-dried, milled, *Salix koriyanagi* (NWC1038) powder (270 mg) was extracted at 50 °C (10 min) in H_2_O: MeOH (80:20, 5 mL). The sample was centrifuged (5 min) and the supernatant transferred to a new tube and heated at 90 °C (2 min). After cooling and centrifugation the supernatant (3.0 mL) was removed to a glass HPLC vial for purification by HPLC peak collection.

### 2.4 Spectroscopic data

*Salicin-7-sulfate* **3**: Yellowish amorphous powder (0.9 mg), [α]_25_^D^ − 32.8 (*c* 0.0367, water), UHPLC-MS: RT 9.90 min, UV λ_max_ 210, 271 nm; *m/z* 365.0549 [M-H]^−^ calc'd for C_13_H_17_O_10_S, 365.0542. ^1^H NMR [600 MHz,(D_2_O:CD_3_OD = 8:2)] δ 3.52 (1H, m, H-4′), 3.59–3.63 (2H, m, H-3′,5′), 3.65 (1H, dd, *J* = 9.3,7.7 Hz, H-2′), 3.76 (1H, dd, *J* = 12.5, 5.7 Hz, H-6′_β_), 3.93 (1H, dd, *J* = 12.5, 2.2 Hz, H-6′_α_), 5.12 (1H, d, *J* = 11.2 Hz, H-7), 5.22 (1H, d, *J* = 11.2 Hz, H-7), 5.10 (1H, d, *J* = 7.6 Hz, H-1′), 7.17 (1H, td, *J* = 7.5, 1.0 Hz, H-4), 7.24 (1H, d, *J* = 8.0 Hz, H-6), 7.43 (1H, td, *J* = 8.3, 1.6 Hz, H-5), 7.48 (1H, dd, *J* = 7.6, 1.6 Hz, H-3). See Table 2 for ^13^C NMR and Supplementary information file for 2D spectra.

## 3 Results and discussion

**Fig. 2:**
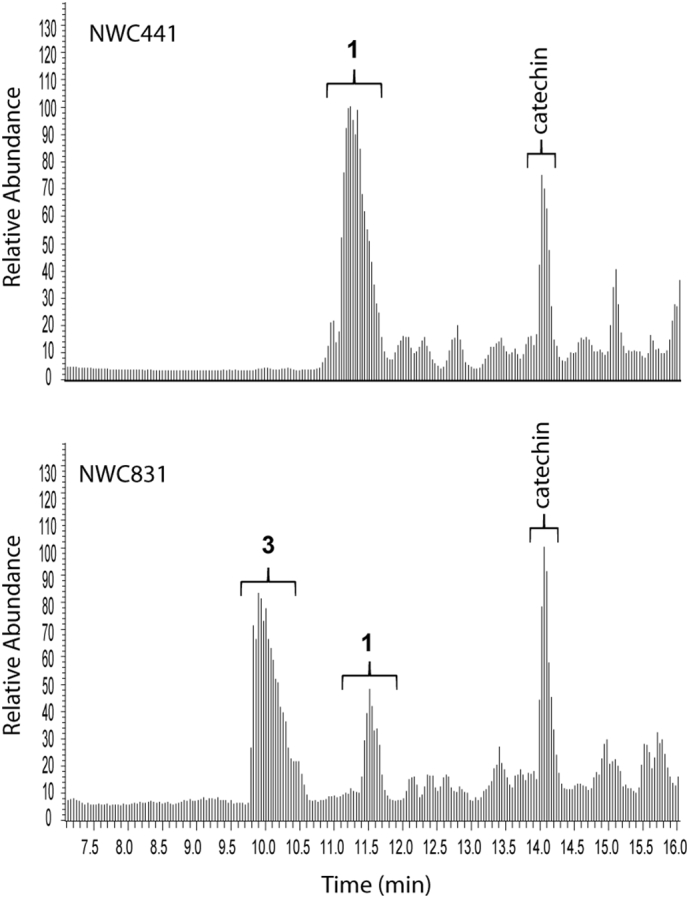
Total Ion Chromatograms (negative ion mode) of two accessions (NWC441 and NWC 831) from the natural willow collection. **1**: salicin, **3**: salicin-7-sulfate.

**Fig. 3:**
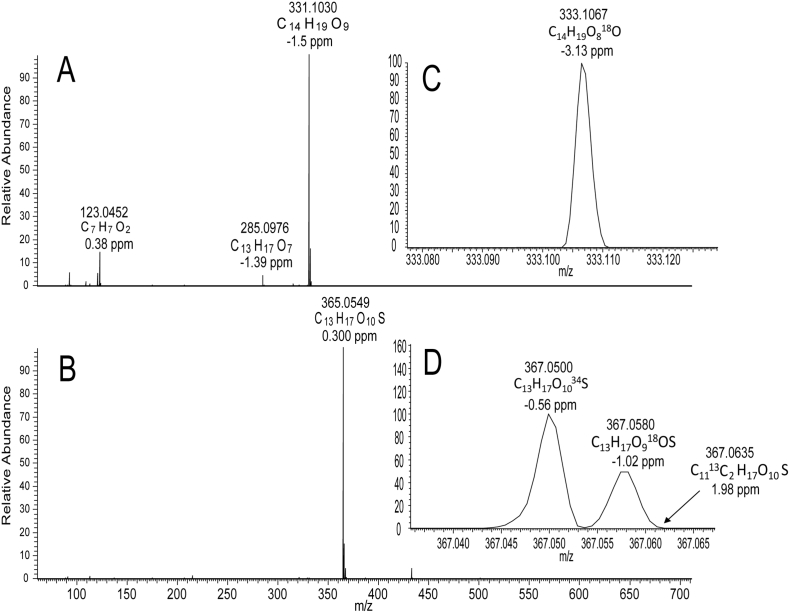
A: MS spectrum of **1** (*m/z* 331, 11.27 min); B: MS spectrum of **3** (*m/z* 365, 9.90 min); C: Enlarged region showing *m/z* 333 ion [(M+2)+formate-H] of **1**; D: Enlarged region of *m/z* 367 ions relating to [(M+2)-H] ions of **3**. Data was collected in negative ion mode.

Data from a standardised ^1^H-NMR fingerprinting method for aqueous methanolic extracts of willow [11], that gives quantitative data on a mixture of primary and secondary metabolites, was mined to examine the variation in salicin concentration in stem tissue samples, across 86 pure (i.e non-hybrid) *Salix* genotypes in the NWC, harvested at the dormant stage (February), a time-point when biomass willows are generally cropped. Quantitative data derived from ^1^H-NMR *via* integration of the distinctive and isolated benzylic hydrogens of salicin **1** (δ4.74 and δ4.69) against internal d_4_- trimethylsilylpropionate standard, are given in Table 1. Salicin levels varied from 2.85 (*S. maccaliana*) to 57.6 (*S. acutifolia* Willd.) mg/g dry weight, (i.e. 0.29% to 5.8% dry weight) of whole stem tissue. Interestingly, the *Salix sp*. (*alba, purpurea, fragilis* and *daphnoides*), that are documented for medicinal use by the Herbal Medicinal Product Committee of European Medicines Agency [12], were not the highest in salicin content, ranging from 0.84 to 3% dry weight. The highest salicin contents were found in *S. acutifolia* (5.76%) and *S. rorida* (4.83%). *S. acutifolia* has previously been found to contain mainly salicin **1** and salicortin **4** in the emerging green shoots during the rapid growth season (May) [13] and this agrees with the finding here that much of the salicin remains in the matured stem tissue in the dormant season. High-resolution LC-MS data (negative ionisation mode) was also collected on comparative extracts of all samples for direct comparison with the ^1^H-NMR data. The peak corresponding to salicin **1** appeared at 11.27 min (Fig. 2) and gave ions at *m/z* 285.0976 (C_13_H_17_O_7_) corresponding to [M-H]^-^ and at *m/z* 331.1030 (C_14_H_19_O_9_) which corresponded to the formate adduct (Fig. 3).

**Table 1:** Concentration of salicin, **1**, in 86 accessions from the National Willow Collection (NWC) held at Rothamsted Research (RRes). Data is obtained from ^1^H-NMR analysis (600 MHz) of a D_2_O:CD_3_OD (4:1) extract of dormant stem tissue.

ID	NWC plot code	RRes no	Species	Variety	Salicin, 1 mg/g d.w.
1	M30	1165	*S. arbusculoides*		15.35 ± 0.08
2	M130	1043	*S. wilsonii*		7.88 ± 1.00
3	M46	1062	*S. rosmarinifolia*		45.04 ± 0.97
4	M147	1236	*S. rhamnifolia* Pall.		8.51 ± 2.41
5	M28	-	*S. waldsteiniana* Willd.		2.88 ± 0.83
6	NWC742	-	*S. bebbiana*		8.14 ± 0.87
7	GH1346	-	*S. kalarica*		11.48 ± 0.14
8	GH1239	1239	*S. saposhnikovii*		6.24 ± 0.68
9	M56	415	*S. magnifica* Hemsl.		8.18 ± 0.21
10	M36	500319	*S. balfourii*		10.90 ± 1.55
11	M164	500340	*S. humilis*	microphylla	19.40 ± 0.26
12	M60	746	*S. gracilistyla* Miq.	Neko-Yanagi	6.83 ± 0.17
13	NWC1011	791	*S. alberti*		12.72 ± 1.98
14	M38	823	*S. caesia* Vill.	Misurina. Belluna	18.15 ± 0.54
15	M39	830	*S. kochiana* Traut.		18.79 ± 0.04
16	M113	888	*S. pychnostachya*		9.01 ± 0.14
17	NWC1096	889	*S. suchowensis*		13.23 ± 0.52
18	NWC1037	828	*S. integra* Thunb.		15.13 ± 0.39
19	M121	984	*S. alaxensis* Anderss.		18.55 ± 1.80
20	M129	-	*S. elaeagnos* Scop.		11.68 ± 1.08
21	M27	-	*S. nakamurana*		4.32 ± 0.22
22	M47	-	*S. hastata* L.		26.87 ± 2.32
23	M54	-	*S. commutata* Bebb		40.93 ± 3.31
24	NWC1231	-	*S. excelsa*		11.07 ± 0.00
25	NWC1306	1317	*S. lasiocarda*	Musgroves Orange	26.11 ± 0.00
26	NWC1308	1339	*S. patula*		3.92 ± 0.00
27	NWC1309	-	*S. tetrapla*		7.32 ± 0.00
28	NWC1315	-	*S. discolor*		6.88 ± 0.00
29	NWC1316	-	*S. ehrhartiana*		25.13 ± 0.00
30	NWC1317	-	*S. exigua*		9.95 ± 0.00
31	NWC1318	-	*S. lasiocarpa*		10.95 ± 0.00
32	NWC1319	-	*S. meyeriana*		28.63 ± 0.00
33	NWC1320	-	*S. pellita*		25.63 ± 0.00
34	NWC1321	-	*S. pendulina*		11.64 ± 0.00
35	NWC1322	-	*S. wardiana*		11.17 ± 0.00
36	NWC695	500579	*S. pyrolifolia*		8.79 ± 0.00
37	NWC1195	1017	*S. phylicifolia* L.		4.12 ± 1.01
38	NWC1196	1018	*S. reinii* Franch. et Savat.		15.17 ± 0.19
39	NWC1165	1024	*S. hookeriana* Barratt ex Hook.		28.16 ± 1.12
40	NWC1202	1038	*S. sitchensis* Sanson ex Bong.		24.40 ± 4.03
41	NWC285	11	*S. lucida* Muhl.		38.53 ± 5.82
42	NWC1214	1130	*S. maccaliana*		2.85 ± 0.05
43	NWC1215	1131	*S. wimmeriana*		21.35 ± 0.01
44	NWC1216	1155	*S. acutifolia* Willd.		57.57 ± 1.88
45	NWC1219	1157	*S. adhenophylla* Hook.		6.95 ± 0.60
46	NWC722	1184	*S. cinerea* L.		8.50 ± 1.39
47	M137	1187	*S. coesia* Vill.		14.45 ± 0.63
48	NWC1236	1208	*S. irrorata* Anderss.		22.91 ± 4.30
49	NWC1237	1209	*S. kangensis* Nakai		37.39 ± 1.45
50	NWC1241	1218	*S. nipponica* (Franch. et Sav.) A.Skvorts.		10.13 ± 1.65
51	NWC1245	1229	*S. pierotii* Miq.		12.73 ± 1.62
52	NWC1301	1285	*S. udensis* Trautv. et Mey.		4.96 ± 0.35
53	NWC1302	1301	*S. vinogradovii* A.Skvorts.		29.87 ± 2.12
54	M162	1308	*S. repens* L.	Argentea	6.13 ± 0.93
55	M93	14	*S. pentandra* L.	Dark French	21.19 ± 3.45
56	NWC470	191	*S. alba* L.	Kew	8.40 ± 0.57
57	NWC279	2	*S. nigra* Marsh.	SN3 Primrose Hill	28.90 ± 4.79
58	NWC488	210	*S. alba* L. var.coerulea	Wantage Hall	12.21 ± 0.62
59	NWC295	23	*S. amygdaloides* Anderss.		20.07 ± 3.95
60	M55	261	*S. babylonica* L.	Annularis	7.96 ± 0.09
61	NWC647	386	*S. fragilis* L.	Cox	19.06 ± 0.65
62	M69	420	*S. daphnoides* Vill.	Ruberrima	24.70 ± 5.93
63	NWC688	441	*S. rorida* Lacksch.		48.26 ± 2.30
64	NWC692	446	*S. aegyptiaca* L.		9.25 ± 0.11
65	NWC698	449	*S. appendiculata* Vill.	Venzonassa	3.57 ± 0.64
66	NWC701	452	*S. apennina* A.Skvorts.	Pescara	26.87 ± 1.47
67	NWC704	455	*S. aurita* L.	Innis Moor	6.67 ± 0.24
68	NWC708	459	*S. caprea* L.	Smithiana	6.31 ± 0.97
69	NWC733	481	*S. scouleriana* Barrat ex Hook.		32.45 ± 0.70
70	NWC326	56	*S. triandra* L. f.concolor	Baldwin	4.24 ± 0.41
71	NWC821	577	*S. dasyclados* Wimm.		6.68 ± 0.01
72	NWC841	607	*S. rehderiana* C.K. Schneider		10.54 ± 3.14
73	NWC849	615	*S. schwerinii* E.Wolf	K3 Hilliers	7.04 ± 0.29
74	NWC890	672	*S. viminalis* L.	Bowles Hybrid	5.01 ± 0.11
75	NWC963	741	*S. turanica* Nasarov		4.76 ± 0.06
76	NWC1032	820	*S. amplexicaulis*	Bory	6.61 ± 1.03
77	NWC1034	824	*S. gilgiana* Seemen		15.12 ± 2.25
78	NWC1038	831	*S. koriyanagi* Kimura ex Goerz		24.16 ± 0.00
79	NWC1053	844	*S. purpurea* L.	Uralensis	29.25 ± 1.56
80	NWC1095	886	*S. caspica* Pall.		14.14 ± 1.89
81	NWC1097	890	*S. tenuijulis* Ledeb.		13.99 ± 2.93
82	NWC1046	941	*S. miyabeana* Seemen	Purpurescens	28.11 ± 1.02
83	NWC1141	956	*S. eriocephala* Michx.	Mawdesley	12.80 ± 0.24
84	NWC1170	988	*S. drummondiana* Barratt ex Hook.		36.28 ± 1.84
85	NWC1174	993	*S. laggerii* Wimm.		7.03 ± 0.23
86	NWC1175	994	*S. mielichhoferii* Saut.	Seiseralp	11.70 ± 0.74

**Fig. 4:**
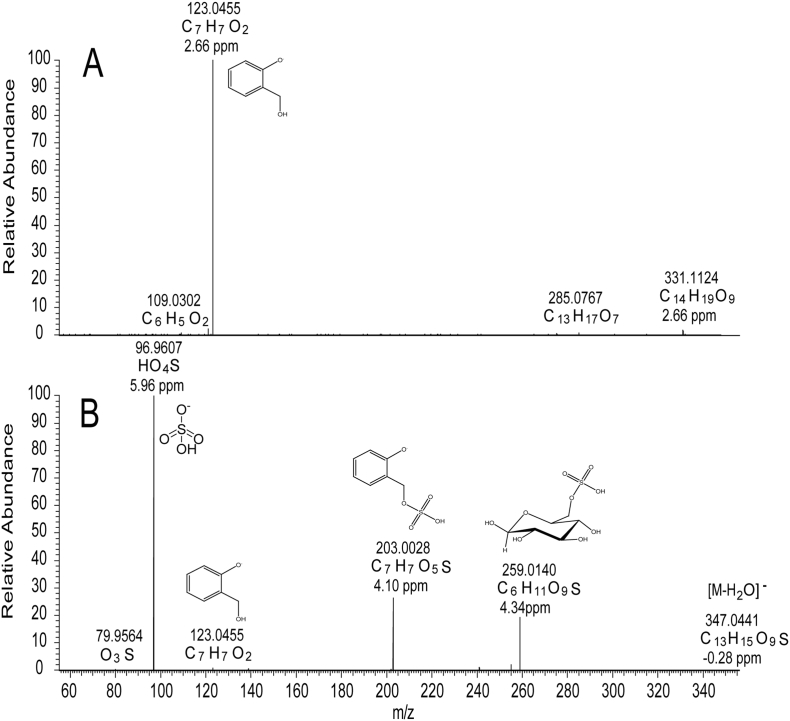
A: MSMS spectrum of **1** (m/z 331, 11.27 min); B: MSMS spectrum of **3** (m/z 365, 9.90 min). Data was collected in negative ion mode.

MSMS of *m/z* 331.1030 gave a single fragment ion at *m/z* 123.0455 corresponding to the C_7_H_7_O_2_ salicyl alcohol aglycone moiety (Fig. 4). In many lines, a further peak corresponding to **3** was present in the same region of the Total Ion Chromatogram appearing at 9.90 min (Fig. 2). The mass spectrum of **3** contained an ion at *m/z* 365.0549 and a formula corresponding to C_13_H_17_O_10_S (Fig. 3). The presence of sulfur in the molecule was confirmed via inspection of the M+2 isotope region. In addition to the ions at *m/z* 367.0580 and *m/z* 367.0635, corresponding the ^18^O (C_13_H_17_O_9_^18^OS) and ^13^C (C_11_^13^C_2_H_17_O_10_S) isotopes respectively, an ion at *m/z* 367.0500 was present and corresponded to an entity with molecular formula C_13_H_17_O_10_^34^S. These M+2 ions and their relative intensities, are consistent for S-containing metabolites when data is collected on MS instruments with a high resolving power (such as FT-ICR-MS or Orbitrap) and have previously been suggested to confirm the molecular formulae of sulfur bearing metabolites [14].

The MSMS spectrum (Fig. 4) showed a base peak at *m/z* 96.9607 [SO_4_H]^−^. Other peaks at *m/z* 347.0441 (C_13_H_15_O_9_S), 203.0028 (C_7_H_7_O_5_S) and 123.0455 (C_7_H_7_O_2_) corresponded to [M-H_2_O]^−^, [M-glucose]^−^ and [salicyl]^−^ respectively. The MS data thus indicated that **3** is a sulfated derivative of salicin. The presence of a further ion at *m/z* 259.0140 (C_6_H_11_O_9_S), corresponding to sulfated glucose, seemed to indicate that the sulfate group was possibly located on the glucose moiety of salicin. However, this ion could also arise from a rearrangement and neutral loss of orthoquinone methide from a 7-sulfate as shown in Fig. S1, Supporting information. Small ions arising from an analogous neutral loss are present in the published MS data of other salicinoids, e.g. a 423 → 317 transition in salicortin **4** that have not, to date, been rationalised [15], but can be explained by a similar loss of orthoquinone methide and transfer of the 7-ester group to glucose. Thus, from MS data alone it was impossible to assign the position of the sulfate group with certainty. The ions obtained in the MS-MS spectrum suggested three possible structures, that of salicin-7-sulfate, isosalicin-1-sulfate (i.e. 7-glucosylsalicyl alcohol-1-sulfate) or salicin-2′/6′-sulfate. The final structure was determined *via* isolation using repeated HPLC injections and structural characterisation by 1D and 2D-NMR.

The ^1^H-NMR (Table 2 and Fig. S2, Supporting information) was compared to that of salicin **1** to determine the position of sulfation. A clear 0.44 ppm downfield shift of the two J = 11 Hz doublet signals relating to the methylene group at C-7 were observed. These signals now appeared at δ5.216 and δ5.123 in contrast to those observed in salicin (δ4.734 and δ4.681). Signals relating to the glucoside moiety were largely unchanged as where those of the aromatic salicyl unit. Thus, the ^1^H-NMR data suggested a structure of salicin-7-sulfate **3**. ^13^C NMR data was obtained from HSQC and HMBC data and is presented in Table 2 and Figs. S4 and S5, supporting information. The most significant difference was a 6.8 ppm downfield shift of the signal relating to C-7 which appeared at 68.8 ppm in comparison to the equivalent carbon in salicin (62.0 ppm). An associated upfield shift of 6.4 ppm was observed for the signal corresponding to C-2 which now appeared at 127.5 ppm. Salicin-7-sulfate **3** has not previously been reported in the literature. However, ^1^H and ^13^C NMR data is available for other sulfated natural products. The structurally related idesin hydrogen sulfate **5** isolated from the fruits of *Idesia polycarpa* Maxim. (Flacourtiaceae) showed comparable changes in its NMR spectra (Table 2) when compared to the non-sulfated compound, i.e. a downfield shift of 0.54 and 5.7 ppm for the protons and carbon, respectively, attached to the sulfate group [16]. Similarly, the position of a sulfate group at C-6 of glucose in a triterpene glycoside, isolated from the whole plant of *Bacopa monnieri* (L.) Wettst. (Scrophulariaceae), was also confirmed based on a downfield shift of 3.4 ppm in the carbon directly attached to the sulfate group [17]. Although sulfation of natural products is not rare, and occurs often in mammalian metabolism, most examples from the plant world concern sulfated flavonoids [18]. Other instances from plant pathways include a sulfated anthraquinone [19] and a sulfate of deoxylactucin – a sesquiterpene from lettuce [20]. This report and that of the related idesin hydrogen sulfate [16] are the first examples from simple phenolic metabolism although there are examples of sulfated lignans (e.g. [21,22]).

**Table 2:** Chemical shift data of salicin **1**, salicin-7-sulfate **3** and idesin hydrogen sulfate 5.

Position	**1**^a^			**3**^a^			**5**^b^, ^c^		
	δ_C_	δ_H_	*J*_H-H_ (Hz); multiplicity^d^	δ_C_	δ_H_	*J*_H-H_ (Hz); multiplicity^d^	δ_C_	δ_H_	*J*_H-H_ (Hz); multiplicity^d^
1	157.6	-	-	157.8	-	-	144.1	-	-
2	133.9	-	-	127.5	-	-	132.0	-	-
3	132.1	7.40	7.5; 1.5; *dd*	133.7	7.48	7.6; 1.6; *dd*	121.1	6.96	7.5; 2.0; *dd*
4	126.0	7.15	7.5; 1.0; *td*	126.1	7.17	7.5; 1.0; *td*	126.6	7.01	7.5; *t*
5	132.3	7.37	8.3; 1.7; *td*	133.7	7.43	8.3; 1.6; *td*	117.6	6.86	7.5; 2.0; *dd*
6	118.0	7.21	8.0; *d*	118.1	7.24	8.0; *d*	150.6	-	-
7	62.0	4.68 4.73	12.7; *d* 12.7; *d*	68.8	5.12 5.22	11.2; *d* 11.2; *d*	66.2	5.32 5.19	12.0; *d* 12.0; *d*
1′	103.4	5.08	7.4; *d*	103.7	5.10	7.6; *d*	106.9	4.63	7.5; *d*
2′	75.7	3.56-3.63	Overlapped	76.2	3.65	7.7; 9.3; *dd*	75.3	3.55	*m*
3′	78.6	3.56-3.63	Overlapped	78.7	3.59-3.63	*m*	77.7	3.45-3.36	*m*
4′	72.1	3.50	*m*	72.5	3.52	9.0, 9.8, *dd*	70.9	3.45-3.36	*m*
5′	78.6	3.56-3.63	*m*	78.7	3.59-3.63	*m*	78.3	3.32	*m*
6′	63.3	3.75 3.91	12.4; 5.7; *dd* 12.4; 2.2; *dd*	63.9	3.76 3.93	12.5; 5.7; *dd* 12.5; 2.2; *dd*	62.1	3.88 3.75	12.3; 2.3; *dd* 12.3; 5.0; *dd*

a Data collected in 80:20 D_2_O:CD_3_OD (4:1). Spectra were referenced to d_4_-TSP at δ0.00.

b Data collected in d_4_-MeOH. Spectra were referenced to d_4_-MeOH.

c Chou et al. [16].

d *d* doublet; *dd* double doublet; *m* multiplet; *dt* doublet of triplets; *t* triplet.

**Fig. 5:**
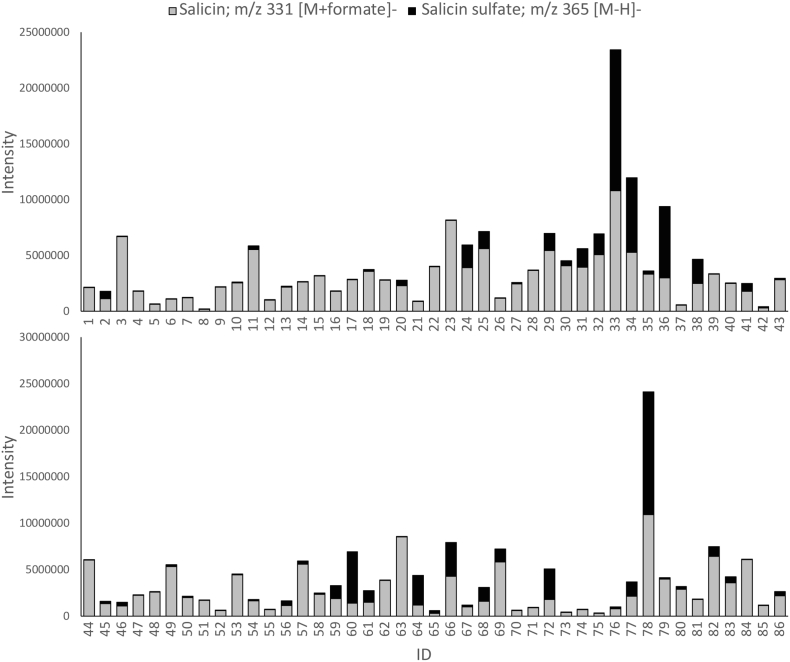
Ion intensities of salicin **1** (m/z 331, grey) and salicin-7-sulfate **3** (m/z 365, black) from negative mode LC-MS data. ID numbers refer to entries in Table 1.

Unlike salicin, it was not possible, due to overlapping signals, to quantify salicin-7-sulfate in the NMR spectra. However, relative quantitation within the original LC-MS datasets arising from the 86 genotypes screened, was used to gain insight into the abundance of the sulfated form (Fig. 5). It can be seen, by correlating the NMR-quantified levels of salicin shown in Table 1 with the relative intensities of the salicin peak in the output data table from Sieve^TM^ software processing of the LC-MS data (see Fig. S6, supporting information for the correlation), that the LC-MS ‘quantitation’ in general shows good correlation with the NMR data, but there are several significant outliers such as lines 33 and 78 where LC-MS data is high, and lines 41 and 44, where LC-MS is giving low readings. The vagaries of differential ionisation and ion suppression are known influences on LC-MS quantitation and thus the data presented in Fig. 5 provides relative levels rather than absolute quantitation provided by NMR. It also should be noted that the sulfated form of salicin ionises much more easily than salicin and thus Fig. 5 serves as a guide to relative levels of the sulfate across samples rather than accurate quantitation. Nevertheless, the LC-MS data indicated that the ratio of salicin: salicin-7-sulfate was not fixed. Many of willow species produced only trace levels of the sulfated form. The highest amounts were observed in *S. pellita* (ID = 33, NWC1320) and *S. koriyanagi* (ID = 78, NWC1038). However, as a general rule across the dataset, the amount of salicin produced does not correlate with the amount of the sulfated form (as observed by LC-MS). For example, *S. commutata* (ID = 23, M54) and *S. rorida* (ID = 63, NWC688) both contained appreciable amounts of salicin, yet only trace amounts of salicin-7-sulfate. In contrast, *S. babylonica* L. var. Annularis (ID = 60, M55) showed low levels of salicin and a much higher proportion of salicin-7-sulfate.

**Fig 6:**
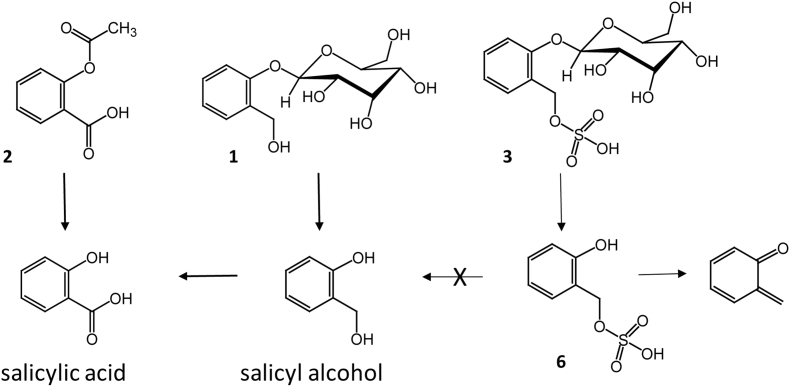
Human metabolism of salicin **1** and aspirin **2** to active salicylic acid and the likely metabolism of salicin-7-sulfate **3** to salicylate analogue **6** and orthoquinone methide.

Although, in many cases the levels of salicin-7-sulfate are very low, the presence of this compound in the varieties that are in both traditional and commercial use as herbal medicines is of concern. Of the above mentioned traditional varieties used in herbal products, S. *alba* and *fragilis,* contained significant amounts of (2) with respect to salicin. The presence of the sulfate group in **3**, when metabolised by humans, is likely to lead to the formation of salicyl alcohol–7-sulfate **6** (Fig. 6) that is unlikely to be further metabolised to salicylate, but more likely to form orthoquinone methide, a reactive entity. There are four pharmacological aspects to consider – (i) that **6** is a close analogue of salicylate and thus itself is a cyclooxygenase inhibitor; (ii) that having the sulfate group gives **6** different anti-coagulant properties (*c.f.* heparin) to salicylate; (iii) that **6** is a much stronger acid than salicylate and thus potentially more harmful in gastro-intestinal bleeding side effects and (iv) that the elimination of sulfuric acid from **6** to form orthoquinone methide *in vivo* may result in co-valent binding to enzyme active sites. Although herbal extracts of *Salix* species also contain other more complex salicinoids, many of these break down to salicin and thus can be considered as further sources of active salicylate. However, the blocking acidic sulfate group means that **3** needs further investigation as this is likely to have direct effects in humans, and may partly explain the different efficacies of herbal willow and synthetic aspirin. Also, from a safety of herbal medicines perspective, the pharmacology of salicin-7-sulfate **3** warrants further investigation, and our efforts are now focussed on obtaining larger quantities of this new analogue for this purpose.

## Conflict of interest

The authors declare no conflict of interest.

## Appendix A Supplementary data

Supplementary material Image 1
