# Supplementary material for: Salicin-7-sulfate: A new salicinoid from willow and implications for herbal medicine
Source: Fitoterapia. 2018 Jun;127:166–72. doi: 10.1016/j.fitote.2018.02.009 (PMC5999357; doi:10.1016/j.fitote.2018.02.009)
Supplement: Supplementary file 1 — Supplementary material [file mmc1.pdf]

Salicin-7-sulfate: a new salicinoid from willow and implications for herbal medicine

Clarice Noleto-Dias, Jane L. Ward, Alice Bellisai, Charlotte Lomax and Michael H. Beale

Supporting Information

Fig. S1. Neutral loss of orthoquinone methide in the MS-MS fragmentation of salicin-7-sulfate **3** and the analogous loss in salicortin **4.**

Fig. S2. ^{1}H-NMR spectrum (80:20 D2O:CD3OD) referenced to d4-TSP (0.01% w/v) collected at 600 MHz. A: salicin; B: salicin sulfate.

Fig. S3. ^{1}H-^{1}H COSY NMR spectrum (80:20 D2O:CD3OD) of salicin-7-sulfate **3** referenced to d4-TSP (0.01% w/v) collected at 600 MHz.

Fig. S4. ^{1}H-^{13}C HSQC NMR spectrum (80:20 D2O:CD3OD) of salicin-7-sulfate **3** referenced to d4-TSP (0.01% w/v).

Fig. S5. Key ^{1}H-^{13}C HMBC correlations observed of isolated salicin-7-sulfate **3.**

Fig. S6. Correlation of Salicin levels (mg/g d.m) obtained *via* ^{1}H-NMR and LC-MS (negative ion mode)

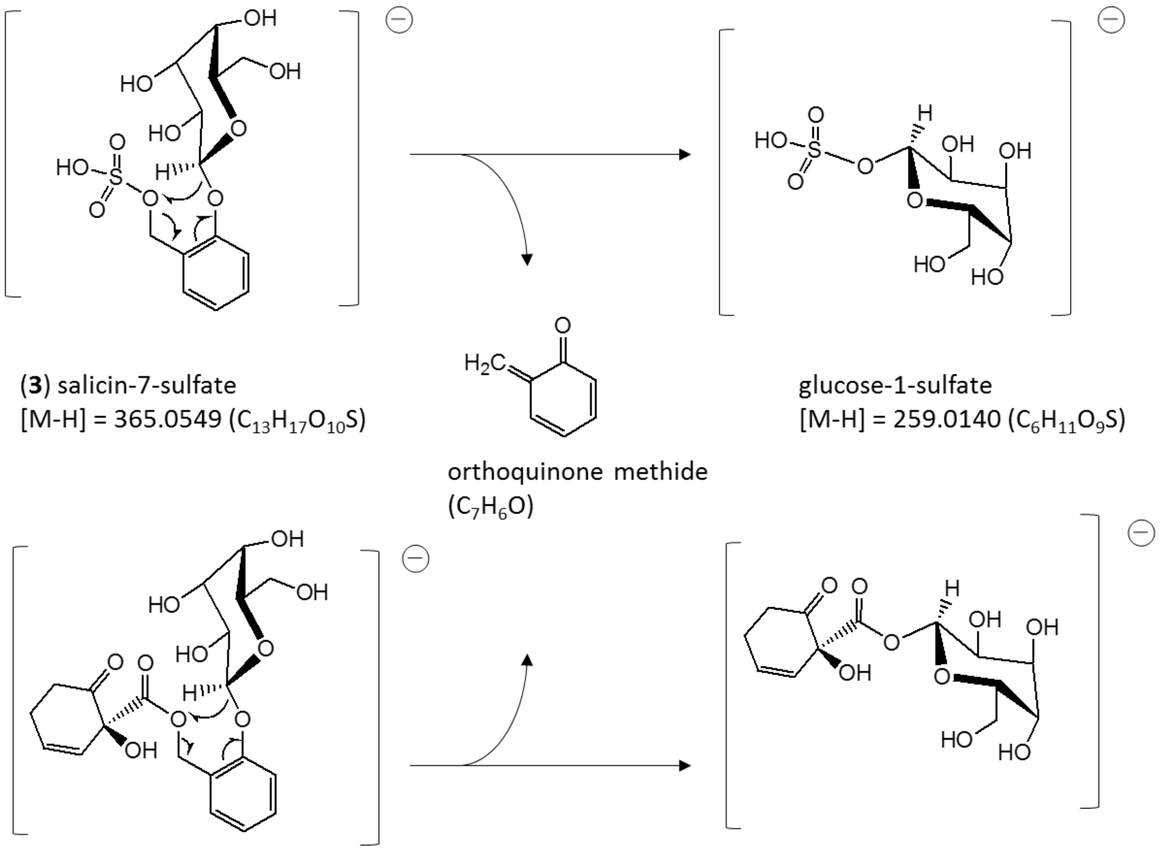

(**3**) salicin-7-sulfate
[M-H] = 365.0549 ($C_{13}H_{17}O_{10}S$)

orthoquinone methide
(C_7H_6O)

glucose-1-sulfate
[M-H] = 259.0140 ($C_6H_{11}O_9S$)

(**4**) salicortin
[M-H] = 423 ($C_{20}H_{23}O_{10}$)

HCC-glucose
[M-H] = 317 ($C_{13}H_{17}O_9$)

Fig. S1. Neutral loss of orthoquinone methide in the MS-MS fragmentation of salicin-7-sulfate **3** and the analogous loss in salicortin **4.**

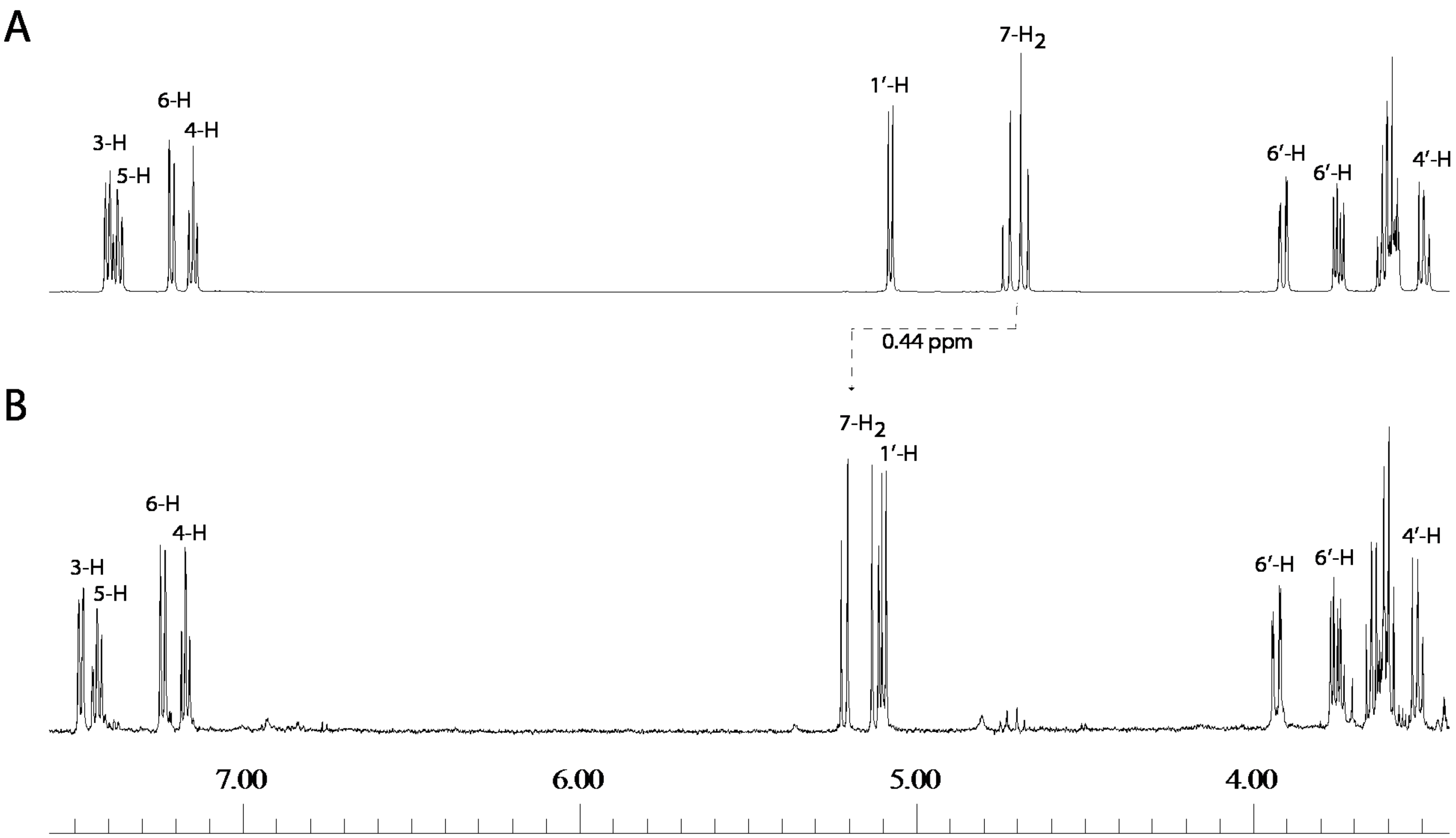

Fig. S2. ^{1}H-NMR spectrum (80:20 D2O:CD3OD) referenced to d4-TSP (0.01% w/v) collected at 600 MHz. A: salicin; B: salicin sulfate.

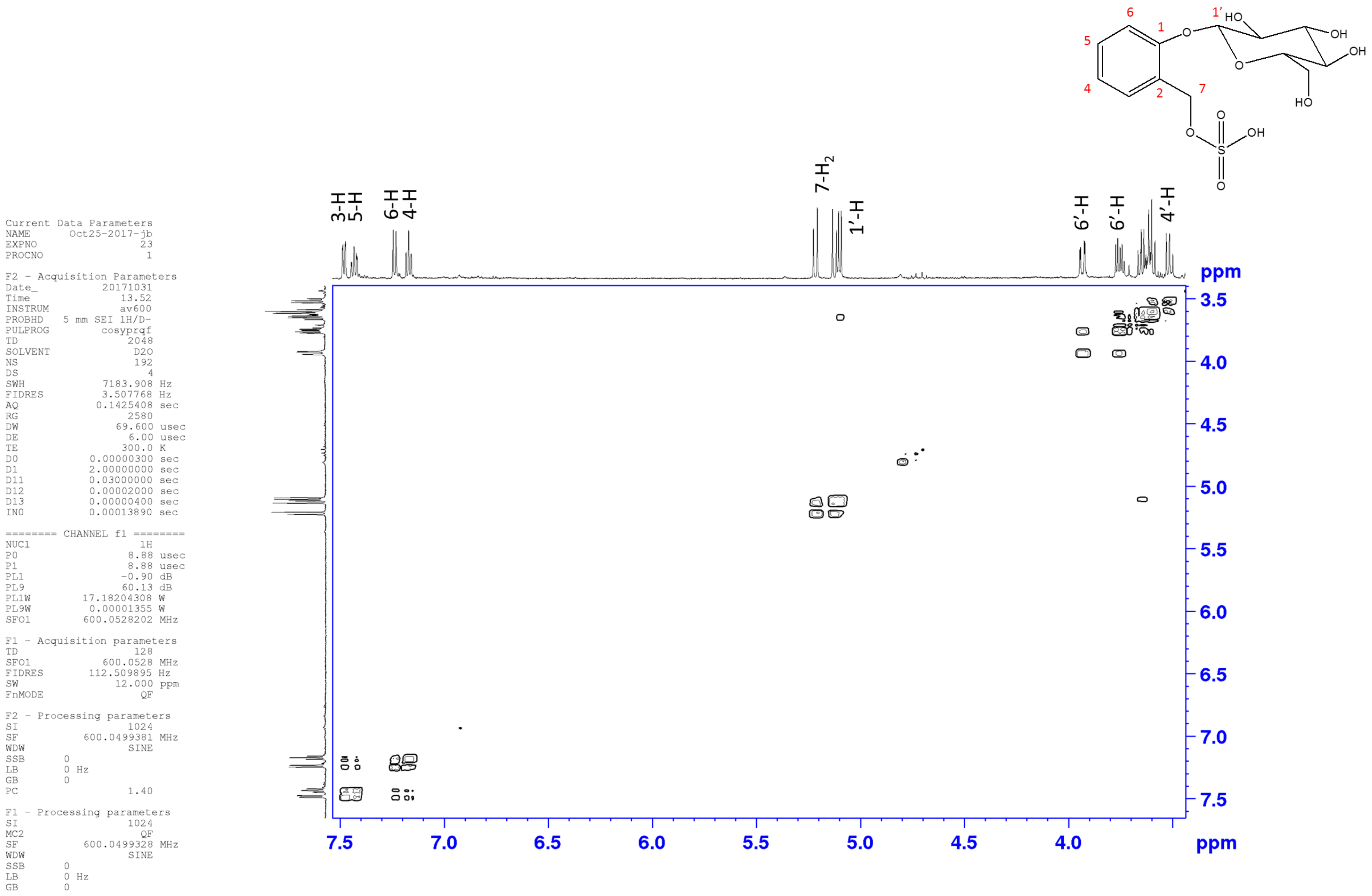

Fig. S3. ^{1}H-^{1}H COSY NMR spectrum (80:20 D2O:CD3OD) of salicin-7-sulfate **3** referenced to d4-TSP (0.01% w/v) collected at 600 MHz.

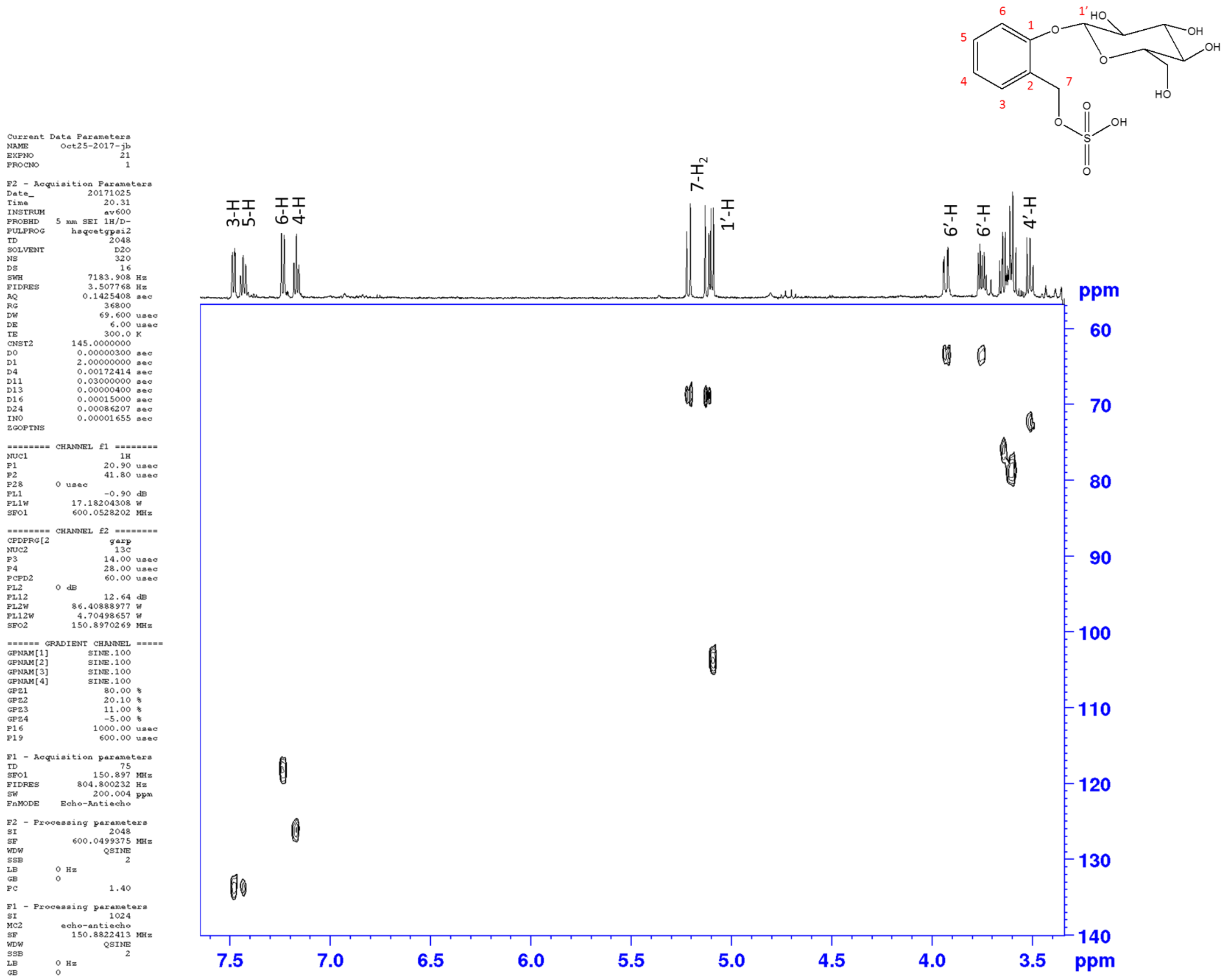

Fig. S4. ^{1}H-^{13}C HSQC NMR spectrum (80:20 D2O:CD3OD) of salicin-7-sulfate **3** referenced to d4-TSP (0.01% w/v).

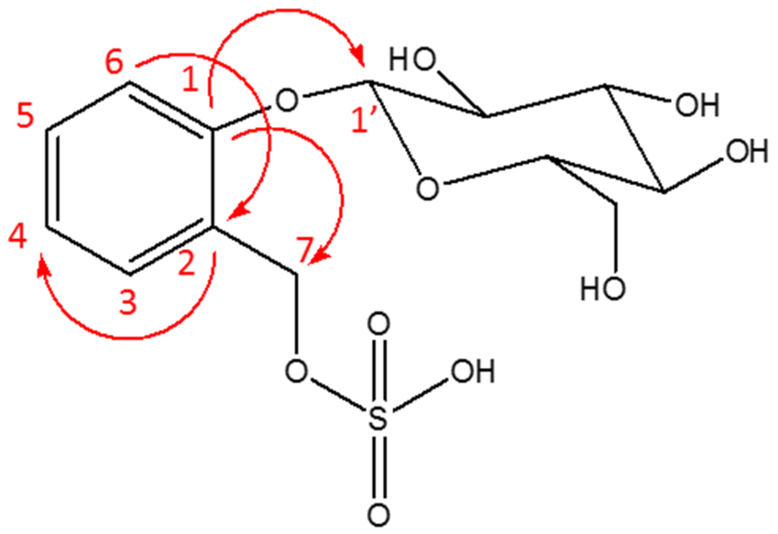

Fig. S5. Key ^{1}H-^{13}C HMBC correlations observed of isolated salicin-7-sulfate **3.**

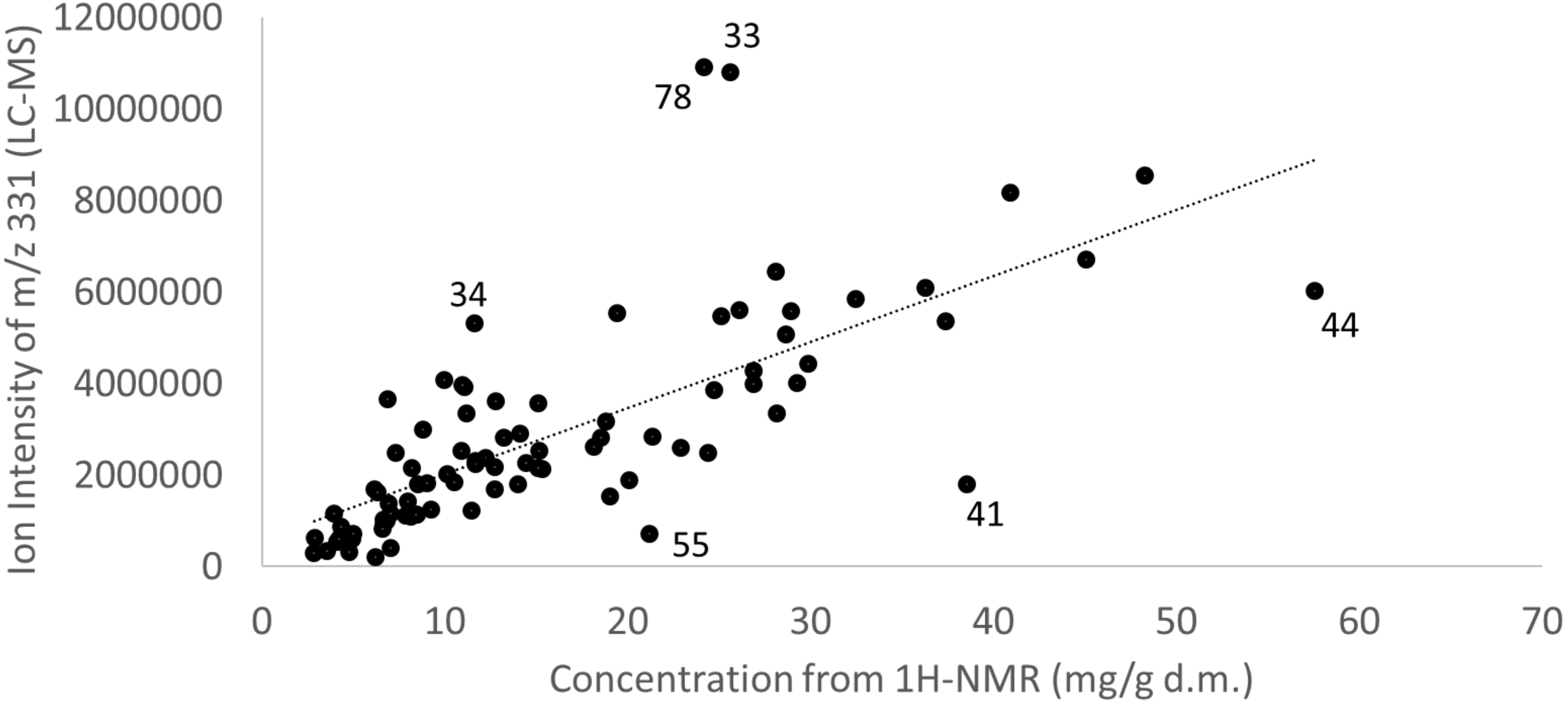

Fig. S6. Correlation of Salicin levels (mg/g d.m) obtained *via* ^{1}H-NMR and LC-MS (negative ion mode). Numbers in the Plot refer to ID numbers in Table 1 (Main Text)
